# Two ortho­rhom­bic polymorphs of hydro­morphone

**DOI:** 10.1107/S2056989016006563

**Published:** 2016-04-26

**Authors:** Jaroslaw Mazurek, Marcel Hoffmann, Ana Fernandez Casares, D. Phillip Cox, Mathew D. Minardi, Josh Sasine

**Affiliations:** a Crystallics B.V., Meibergdreef 31, 1105 AZ Amsterdam, The Netherlands; b Noramco Inc., 503 Carr Rd, Suite 200, Wilmington, DE 19809, USA; c Noramco Inc., 1440 Olympic Drive, Athens, GA 30601, USA

**Keywords:** crystal structure, polymorphism, hydro­morphone,hydrogen bonding

## Abstract

Conditions to obtain two polymorphic forms by crystallization from solution were determined for the analgesic drug hydro­morphone [C_17_H_19_NO_3_; systematic name: (4*R*,4a*R*,7a*R*,12b*S*)-9-hy­droxy-3-methyl-1,2,4,4a,5,6,7a,13-octa­hydro-4,12-methano­benzofuro[3,2-*e*]iso­quinolin-7-one]. These two crystalline forms, designated as I and II, belong to the *P*2_1_2_1_2_1_ ortho­rhom­bic space group. In both polymorphs, the hydro­morphone mol­ecules adopt very similar conformations with some small differences observed only in the *N*-methyl amine part of the mol­ecule. The crystal structures of both polymorphs feature chains of mol­ecules connected by hydrogen bonds; however, in form I this inter­action occurs between the hydroxyl group and the tertiary amine N atom whereas in form II the hydroxyl group acts as a donor of a hydrogen bond to the O atom from the cyclic ether part.

## Abstract 2

Conditions to obtain two polymorphic forms by crystallization from solution were determined for the analgestic drug hydro­morphone. In both polymorphs, the hydro­morphone mol­ecules adopt very similar conformations with some small differences observed only in the *N*-methyl amine part of the mol­ecule. The crystal structures of both polymorphs feature chains of mol­ecules connected by hydrogen bonds

## Chemical context   

Drug polymorphism has been the subject of hundreds of publications and numerous excellent reviews (Byrn *et al.*, 1999 ▸; Grant, 1999 ▸; Singhal & Curatolo, 2004 ▸; Vippagunta *et al.*, 2001 ▸). It is well established that polymorphs with different stability may have different solubility and dissolution rates, which can affect the bioavailability. The semi-synthetic opiate drug hydro­morphone is a potent derivative of morphine and despite poor bioavailability (Parab *et al.*, 1988 ▸) is commonly used to treat moderate to severe pain in the treatment of cancer (Sarhill *et al.*, 2001 ▸). To improve bioavailability of this compound a polymorph screen was performed that resulted in two solvent-free forms, designated as form I and form II.
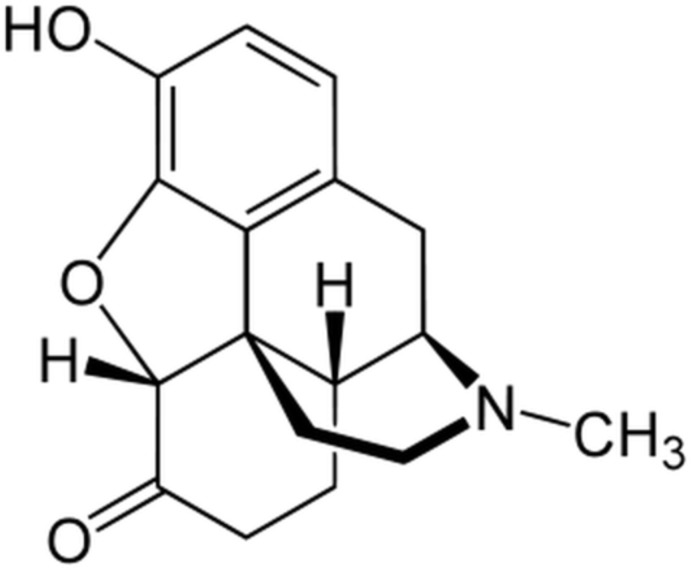


## Structural commentary   

**Figure 1:**
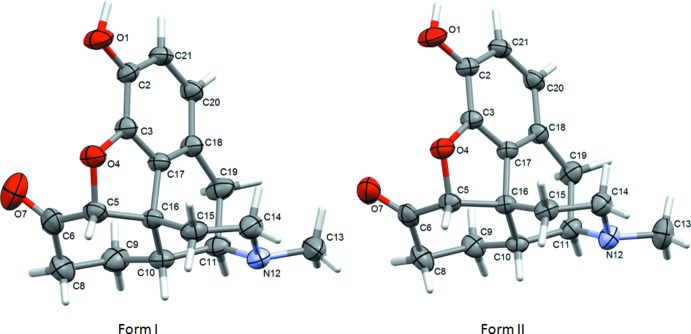
Mol­ecular structure and atom-numbering scheme for hydro­morphone in the crystals of form I (left) and form II (right). Displacement ellipsoids are shown at the 50% probability level.

**Figure 2:**
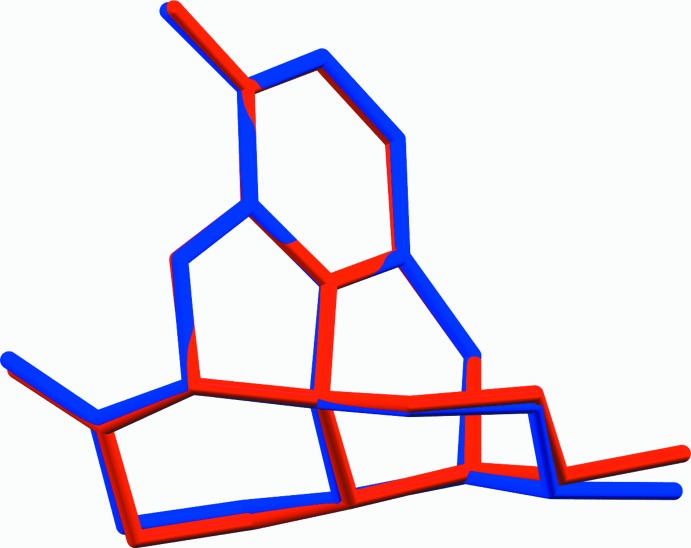
Superposition of the hydro­morphone mol­ecules from two polymorphic forms (red form I, blue form II) generated by fitting of the aromatic ring.

The mol­ecular structure of hydro­morphone in both polymorphs is nearly identical (Fig. 1 ▸) with some deviations found only for the *N*-methyl amine part of the piperidine fragment (Fig. 2 ▸). For example the C10—C11—N12—C13 torsion angle is 178.5 (2)° for form I and 169.5 (2)° for form II. The adopted conformation is similar to the conformation observed for morphine (Bye, 1976 ▸; Scheins *et al.*, 2005 ▸).

## Supra­molecular features   

**Figure 3:**
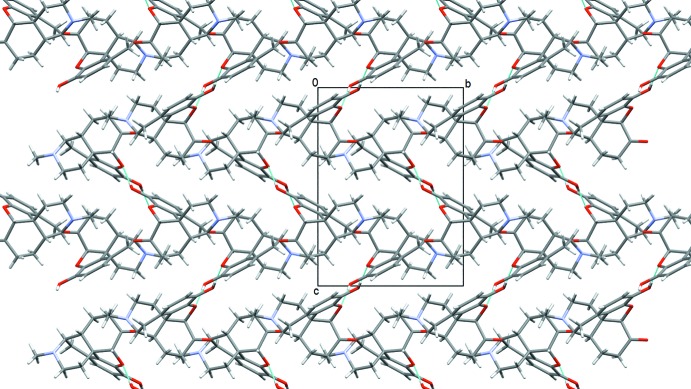
Crystal packing diagram of form I, viewed along the *a* axis. Hydrogen bonds are shown as blue lines.

**Figure 5:**
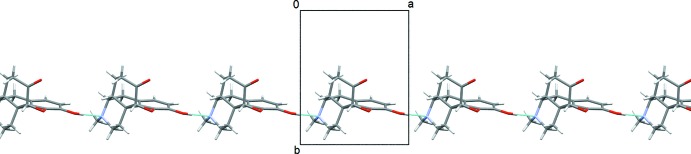
The chain of mol­ecules running along the *a* axis formed by O—H⋯N hydrogen bonds in form I.

**Figure 6:**
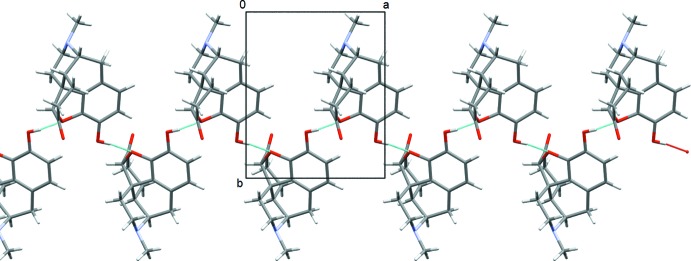
The zigzag chain of mol­ecules running along the *a* axis formed by O—H⋯O hydrogen bonds in form II.

Although both polymorphs crystallize in the same space group *P*2_1_2_1_2_1_ with the same number of mol­ecules in the asymmetric unit, they differ significantly in the packing features (Figs. 3 ▸ and 4 ▸). In form I, the hydrogen-bonded mol­ecules are arranged into chains that run along the *a* axis with adjacent mol­ecules in the chain related by translation. The hydroxyl group donates a hydrogen atom which is accepted by the free electron pair of the N atom (Fig. 5 ▸, Table 1 ▸). In the crystals of form II, inter­molecular hydrogen bonds also generate a chain of mol­ecules that propagates along the *a* axis; however, adjacent mol­ecules along this chain are related by a 2_1_ symmetry axis. The mol­ecules are connected by O—H⋯O hydrogen bonds with the hydroxyl group as donor and the etheric O atom as acceptor (Table 2 ▸). These chains form a zigzag pattern, as illustrated in Fig. 6 ▸. The packing arrangement of mol­ecules in form II is more dense than in polymorph I, as indicated by the Kitajgorodskij (1973 ▸) packing coefficients of 0.71 and 0.69, respectively.

## Synthesis and crystallization   

10.8 mg of hydro­morphone was dissolved in 1.8 mL THF/acetone (1/1, *v*/*v*) and left to evaporate slowly under ambient conditions. After several days, colorless prism-like crystals of form I (m.p. 549.8 K) appeared that were used for diffraction studies. Crystals of form II were obtained in the following way: 19.7 mg of hydro­morphone was suspended in 0.3 mL of 50/50 mixture of ethanol and toluene. The suspension was heated to 333 K and stirred for about one h until it became clear. Subsequently, the vial was cooled rapidly to 278 K and colorless block-like crystals (m.p. 550.2 K) precipitated that were used for diffraction studies.

## Refinement   

The H atoms from the methyl group in form II were included from geometry and their isotropic displacement parameters refined. The remaining H atoms were found in a Fourier difference map and freely refined. The absolute configuration of hydro­morphone was known from the synthetic route. In the absence of significant anomalous scattering effects, Friedel pairs were merged. Crystal data, data collection and structure refinement details are summarized in Table 3 ▸.

## Supplementary Material

Crystal structure: contains datablock(s) I, II. DOI: 10.1107/S2056989016006563/gk2659sup1.cif

Structure factors: contains datablock(s) I. DOI: 10.1107/S2056989016006563/gk2659Isup2.hkl

Supporting information file. DOI: 10.1107/S2056989016006563/gk2659Isup4.mol

Structure factors: contains datablock(s) II. DOI: 10.1107/S2056989016006563/gk2659IIsup3.hkl

Supporting information file. DOI: 10.1107/S2056989016006563/gk2659IIsup5.mol

CCDC references: 1474753, 1474752

Additional supporting information:  crystallographic information; 3D view; checkCIF report

## Figures and Tables

**Figure 4:**
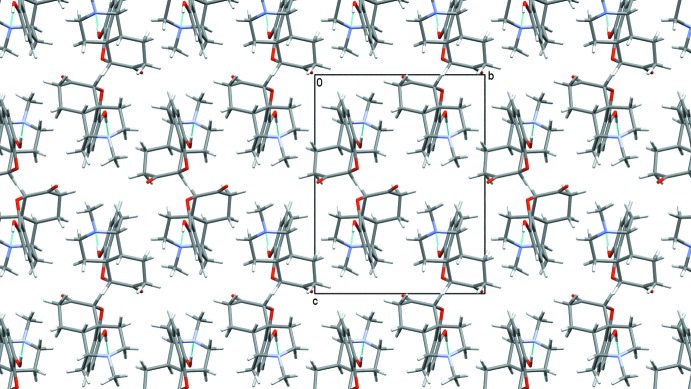
Crystal packing diagram of form II, viewed along the *a* axis. Hydrogen bonds are shown as blue lines.

**Table 1:** Hydrogen-bond geometry (Å, °) for (I)

*D*—H⋯*A*	*D*—H	H⋯*A*	*D*⋯*A*	*D*—H⋯*A*
O1—H1*A*⋯N12^i^	0.91 (4)	1.89 (4)	2.796 (3)	171 (3)

Symmetry code: (i) 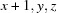.

**Table 2:** Hydrogen-bond geometry (Å, °) for (II)

*D*—H⋯*A*	*D*—H	H⋯*A*	*D*⋯*A*	*D*—H⋯*A*
O1—H1⋯O4^i^	0.84 (3)	1.96 (3)	2.791 (2)	167 (3)

Symmetry code: (i) 
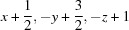
.

**Table 3:** Experimental details

	(I)	(II)
Crystal data		
Chemical formula	C_17_H_19_NO_3_	C_17_H_19_NO_3_
*M*_r_	285.33	285.33
Crystal system, space group	Orthorhombic, *P*2_1_2_1_2_1_	Orthorhombic, *P*2_1_2_1_2_1_
Temperature (K)	296	296
*a*, *b*, *c* (Å)	8.9497 (6), 11.0906 (6), 14.2608 (9)	8.8802 (6), 10.6208 (8), 14.4733 (9)
*V* (Å^3^)	1415.49 (15)	1365.05 (16)
*Z*	4	4
Radiation type	Mo *K*α	Mo *K*α
μ (mm^−1^)	0.09	0.10
Crystal size (mm)	0.35 × 0.35 × 0.30	0.40 × 0.32 × 0.22

Data collection		
Diffractometer	Bruker KappaCCD	Bruker KappaCCD
Absorption correction	–	–
No. of measured, independent and observed [*I* > 2σ(*I*)] reflections	7054, 3427, 3088	15227, 4920, 4693
*R*_int_	0.031	0.022
(sin θ/λ)_max_ (Å^−1^)	0.671	0.758

Refinement		
*R*[*F* ^2^ > 2σ(*F* ^2^)], *wR*(*F* ^2^), *S*	0.042, 0.096, 1.05	0.033, 0.095, 1.07
No. of reflections	3427	4920
No. of parameters	266	257
H-atom treatment	All H-atom parameters refined	H atoms treated by a mixture of independent and constrained refinement
Δρ_max_, Δρ_min_ (e Å^−3^)	0.19, −0.17	0.27, −0.12

Computer programs: *COLLECT* (Hooft, 1998 ▸), *HKL* *DENZO* and *SCALEPACK* (Otwinowski & Minor, 1997 ▸), *SHELXT* (Sheldrick, 2015*a* ▸), *SHELXL2014/7* (Sheldrick, 2015*b* ▸), *Mercury* (Macrae *et al.*, 2006 ▸) and *enCIFer* (Allen *et al.*, 2004 ▸).

## supplementary crystallographic information

### (I) (4R,4aR,7aR,12bS)-9-Hydroxy-3-methyl-1,2,4,4a,5,6,7a,13-octahydro-4,12-methanobenzofuro[3,2-e]isoquinolin-7-one] Crystal data

**Table:**

C_17_H_19_NO_3_	*D*_x_ = 1.339 Mg m^−^^3^
*M**_r_* = 285.33	Melting point < 549.8 K
Orthorhombic, *P*2_1_2_1_2_1_	Mo *K*α radiation, λ = 0.71073 Å
*a* = 8.9497 (6) Å	Cell parameters from 9169 reflections
*b* = 11.0906 (6) Å	θ = 1.0–32.6°
*c* = 14.2608 (9) Å	µ = 0.09 mm^−^^1^
*V* = 1415.49 (15) Å^3^	*T* = 296 K
*Z* = 4	Prism, colorless
*F*(000) = 608	0.35 × 0.35 × 0.30 mm

### (I) (4R,4aR,7aR,12bS)-9-Hydroxy-3-methyl-1,2,4,4a,5,6,7a,13-octahydro-4,12-methanobenzofuro[3,2-e]isoquinolin-7-one] Data collection

**Table:**

Bruker KappaCCD diffractometer	3088 reflections with *I* > 2σ(*I*)
Radiation source: fine-focus sealed tube	*R*_int_ = 0.031
Horizonally mounted graphite crystal monochromator	θ_max_ = 28.5°, θ_min_ = 3.4°
CCD scans	*h* = −11→11
7054 measured reflections	*k* = −11→14
3427 independent reflections	*l* = −17→19

### (I) (4R,4aR,7aR,12bS)-9-Hydroxy-3-methyl-1,2,4,4a,5,6,7a,13-octahydro-4,12-methanobenzofuro[3,2-e]isoquinolin-7-one] Refinement

**Table:**

Refinement on *F*^2^	Primary atom site location: difference Fourier map
Least-squares matrix: full	Secondary atom site location: difference Fourier map
*R*[*F*^2^ > 2σ(*F*^2^)] = 0.042	Hydrogen site location: difference Fourier map
*wR*(*F*^2^) = 0.096	All H-atom parameters refined
*S* = 1.05	*w* = 1/[σ^2^(*F*_o_^2^) + (0.0361*P*)^2^ + 0.2726*P*] where *P* = (*F*_o_^2^ + 2*F*_c_^2^)/3
3427 reflections	(Δ/σ)_max_ = 0.005
266 parameters	Δρ_max_ = 0.19 e Å^−^^3^
0 restraints	Δρ_min_ = −0.17 e Å^−^^3^

### (I) (4R,4aR,7aR,12bS)-9-Hydroxy-3-methyl-1,2,4,4a,5,6,7a,13-octahydro-4,12-methanobenzofuro[3,2-e]isoquinolin-7-one] Special details

**Table:**

Geometry. All esds (except the esd in the dihedral angle between two l.s. planes) are estimated using the full covariance matrix. The cell esds are taken into account individually in the estimation of esds in distances, angles and torsion angles; correlations between esds in cell parameters are only used when they are defined by crystal symmetry. An approximate (isotropic) treatment of cell esds is used for estimating esds involving l.s. planes.

### (I) (4R,4aR,7aR,12bS)-9-Hydroxy-3-methyl-1,2,4,4a,5,6,7a,13-octahydro-4,12-methanobenzofuro[3,2-e]isoquinolin-7-one] Fractional atomic coordinates and isotropic or equivalent isotropic displacement parameters (Å^2^)

**Table:**

	*x*	*y*	*z*	*U*_iso_*/*U*_eq_	
O1	0.91711 (18)	0.7664 (2)	0.17051 (12)	0.0513 (5)	
H1A	0.993 (4)	0.774 (3)	0.213 (2)	0.069 (10)*	
C2	0.7893 (2)	0.7337 (2)	0.21531 (15)	0.0337 (4)	
C3	0.6543 (2)	0.73164 (19)	0.16755 (13)	0.0318 (4)	
O4	0.63079 (17)	0.74990 (16)	0.07223 (10)	0.0409 (4)	
C5	0.4813 (3)	0.7006 (2)	0.05538 (15)	0.0369 (5)	
H5A	0.439 (3)	0.745 (2)	0.0007 (18)	0.034 (6)*	
C6	0.4906 (3)	0.5664 (3)	0.03355 (16)	0.0455 (6)	
O7	0.6063 (3)	0.5185 (2)	0.00966 (16)	0.0692 (6)	
C8	0.3484 (4)	0.4980 (3)	0.0485 (2)	0.0555 (7)	
H8A	0.362 (4)	0.413 (3)	0.031 (2)	0.073 (10)*	
H8B	0.271 (4)	0.541 (3)	0.006 (2)	0.063 (9)*	
C9	0.3038 (3)	0.5078 (2)	0.1523 (2)	0.0464 (6)	
H9A	0.387 (4)	0.480 (3)	0.193 (2)	0.057 (8)*	
H9B	0.220 (3)	0.455 (3)	0.166 (2)	0.055 (8)*	
C10	0.2671 (2)	0.6384 (2)	0.17446 (16)	0.0334 (4)	
H10A	0.175 (3)	0.660 (2)	0.1390 (16)	0.034 (6)*	
C11	0.2315 (2)	0.6636 (2)	0.27875 (16)	0.0365 (5)	
H11A	0.147 (3)	0.610 (2)	0.2993 (18)	0.043 (7)*	
N12	0.1698 (2)	0.78799 (19)	0.28457 (13)	0.0381 (4)	
C13	0.1274 (3)	0.8219 (3)	0.3807 (2)	0.0555 (7)	
H13A	0.072 (4)	0.750 (3)	0.410 (2)	0.068 (9)*	
H13B	0.216 (4)	0.844 (3)	0.417 (2)	0.054 (8)*	
H13C	0.060 (4)	0.893 (3)	0.373 (2)	0.073 (10)*	
C14	0.2738 (3)	0.8808 (2)	0.24843 (19)	0.0426 (6)	
H14A	0.221 (3)	0.956 (3)	0.251 (2)	0.051 (8)*	
H14B	0.359 (3)	0.888 (2)	0.291 (2)	0.046 (7)*	
C15	0.3324 (3)	0.8528 (2)	0.15154 (17)	0.0377 (5)	
H15A	0.251 (3)	0.862 (3)	0.105 (2)	0.048 (7)*	
H15B	0.414 (3)	0.912 (2)	0.1341 (18)	0.043 (7)*	
C16	0.3942 (2)	0.72389 (19)	0.14723 (13)	0.0291 (4)	
C17	0.5225 (2)	0.70844 (19)	0.21381 (14)	0.0289 (4)	
C18	0.5145 (2)	0.6740 (2)	0.30675 (14)	0.0315 (4)	
C19	0.3642 (2)	0.6356 (3)	0.34492 (17)	0.0417 (5)	
H19A	0.364 (3)	0.548 (3)	0.356 (2)	0.058 (9)*	
H19B	0.341 (3)	0.672 (3)	0.406 (2)	0.058 (8)*	
C20	0.6499 (2)	0.6686 (2)	0.35428 (14)	0.0335 (4)	
H20A	0.655 (3)	0.640 (2)	0.4198 (18)	0.039 (6)*	
C21	0.7821 (2)	0.6994 (2)	0.30952 (15)	0.0350 (5)	
H21A	0.871 (3)	0.693 (2)	0.3430 (17)	0.038 (6)*	

### (I) (4R,4aR,7aR,12bS)-9-Hydroxy-3-methyl-1,2,4,4a,5,6,7a,13-octahydro-4,12-methanobenzofuro[3,2-e]isoquinolin-7-one] Atomic displacement parameters (Å^2^)

**Table:**

	*U*^11^	*U*^22^	*U*^33^	*U*^12^	*U*^13^	*U*^23^
O1	0.0239 (8)	0.0907 (15)	0.0393 (9)	−0.0080 (8)	0.0014 (7)	0.0059 (9)
C2	0.0235 (9)	0.0427 (12)	0.0349 (10)	0.0002 (9)	0.0009 (8)	−0.0005 (9)
C3	0.0293 (10)	0.0393 (11)	0.0269 (9)	−0.0021 (8)	0.0004 (8)	0.0035 (8)
O4	0.0315 (8)	0.0656 (11)	0.0256 (7)	−0.0051 (7)	0.0000 (6)	0.0068 (7)
C5	0.0321 (10)	0.0511 (13)	0.0276 (9)	−0.0006 (10)	−0.0047 (9)	0.0035 (9)
C6	0.0490 (14)	0.0573 (15)	0.0301 (10)	0.0061 (12)	−0.0004 (11)	−0.0045 (10)
O7	0.0662 (14)	0.0728 (14)	0.0687 (14)	0.0171 (11)	0.0256 (11)	−0.0007 (11)
C8	0.0547 (16)	0.0505 (16)	0.0615 (17)	−0.0015 (14)	−0.0071 (14)	−0.0212 (14)
C9	0.0390 (13)	0.0362 (12)	0.0640 (16)	−0.0067 (10)	−0.0001 (12)	−0.0024 (11)
C10	0.0262 (9)	0.0360 (11)	0.0380 (11)	−0.0030 (8)	−0.0044 (9)	0.0015 (9)
C11	0.0257 (10)	0.0438 (12)	0.0398 (11)	−0.0059 (9)	0.0004 (9)	0.0046 (10)
N12	0.0266 (8)	0.0487 (11)	0.0389 (9)	−0.0015 (8)	0.0005 (8)	−0.0058 (8)
C13	0.0374 (13)	0.084 (2)	0.0448 (14)	−0.0071 (15)	0.0034 (12)	−0.0195 (14)
C14	0.0357 (12)	0.0383 (13)	0.0536 (14)	0.0002 (10)	0.0000 (11)	−0.0055 (10)
C15	0.0339 (11)	0.0353 (11)	0.0438 (12)	0.0003 (9)	−0.0037 (10)	0.0064 (9)
C16	0.0257 (9)	0.0340 (10)	0.0275 (9)	−0.0015 (8)	−0.0034 (8)	0.0024 (8)
C17	0.0247 (9)	0.0340 (10)	0.0281 (9)	−0.0015 (8)	−0.0033 (8)	0.0024 (8)
C18	0.0281 (9)	0.0391 (11)	0.0274 (9)	−0.0019 (8)	0.0000 (8)	0.0040 (8)
C19	0.0291 (11)	0.0599 (15)	0.0363 (12)	−0.0029 (10)	0.0027 (10)	0.0144 (11)
C20	0.0335 (11)	0.0414 (11)	0.0256 (9)	−0.0002 (9)	−0.0037 (8)	0.0035 (8)
C21	0.0260 (9)	0.0441 (12)	0.0350 (10)	−0.0003 (9)	−0.0074 (9)	−0.0006 (9)

### (I) (4R,4aR,7aR,12bS)-9-Hydroxy-3-methyl-1,2,4,4a,5,6,7a,13-octahydro-4,12-methanobenzofuro[3,2-e]isoquinolin-7-one] Geometric parameters (Å, º)

**Table:**

O1—C2	1.360 (3)	C11—C19	1.548 (3)
O1—H1A	0.91 (4)	C11—H11A	1.01 (3)
C2—C3	1.387 (3)	N12—C13	1.472 (3)
C2—C21	1.398 (3)	N12—C14	1.480 (3)
C3—C17	1.376 (3)	C13—H13A	1.03 (4)
C3—O4	1.390 (2)	C13—H13B	0.98 (3)
O4—C5	1.465 (3)	C13—H13C	1.00 (4)
C5—C6	1.523 (4)	C14—C15	1.510 (3)
C5—C16	1.546 (3)	C14—H14A	0.96 (3)
C5—H5A	1.00 (3)	C14—H14B	0.98 (3)
C6—O7	1.212 (3)	C15—C16	1.534 (3)
C6—C8	1.498 (4)	C15—H15A	0.99 (3)
C8—C9	1.537 (4)	C15—H15B	1.01 (3)
C8—H8A	0.99 (3)	C16—C17	1.500 (3)
C8—H8B	1.03 (3)	C17—C18	1.381 (3)
C9—C10	1.519 (3)	C18—C20	1.390 (3)
C9—H9A	1.00 (3)	C18—C19	1.512 (3)
C9—H9B	0.97 (3)	C19—H19A	0.99 (3)
C10—C16	1.531 (3)	C19—H19B	0.98 (3)
C10—C11	1.546 (3)	C20—C21	1.387 (3)
C10—H10A	1.00 (2)	C20—H20A	0.99 (3)
C11—N12	1.488 (3)	C21—H21A	0.93 (3)
			
C2—O1—H1A	110 (2)	C14—N12—C11	113.08 (18)
O1—C2—C3	120.43 (18)	N12—C13—H13A	108.1 (19)
O1—C2—C21	124.26 (19)	N12—C13—H13B	110.6 (17)
C3—C2—C21	115.31 (18)	H13A—C13—H13B	111 (3)
C17—C3—C2	120.96 (17)	N12—C13—H13C	105 (2)
C17—C3—O4	111.48 (17)	H13A—C13—H13C	111 (3)
C2—C3—O4	127.56 (18)	H13B—C13—H13C	110 (3)
C3—O4—C5	104.13 (15)	N12—C14—C15	113.2 (2)
O4—C5—C6	110.4 (2)	N12—C14—H14A	106.6 (17)
O4—C5—C16	105.03 (16)	C15—C14—H14A	112.8 (17)
C6—C5—C16	111.35 (19)	N12—C14—H14B	109.2 (16)
O4—C5—H5A	106.7 (14)	C15—C14—H14B	108.4 (16)
C6—C5—H5A	110.2 (14)	H14A—C14—H14B	106 (2)
C16—C5—H5A	113.0 (14)	C14—C15—C16	110.72 (18)
O7—C6—C8	122.9 (3)	C14—C15—H15A	109.5 (16)
O7—C6—C5	122.2 (3)	C16—C15—H15A	109.6 (17)
C8—C6—C5	114.8 (2)	C14—C15—H15B	110.0 (15)
C6—C8—C9	108.8 (2)	C16—C15—H15B	109.6 (15)
C6—C8—H8A	110 (2)	H15A—C15—H15B	107 (2)
C9—C8—H8A	110 (2)	C17—C16—C10	109.73 (16)
C6—C8—H8B	104.6 (18)	C17—C16—C15	110.92 (17)
C9—C8—H8B	110.7 (18)	C10—C16—C15	107.40 (17)
H8A—C8—H8B	112 (3)	C17—C16—C5	97.53 (16)
C10—C9—C8	108.9 (2)	C10—C16—C5	119.10 (18)
C10—C9—H9A	109.6 (18)	C15—C16—C5	111.80 (17)
C8—C9—H9A	110.5 (18)	C3—C17—C18	123.80 (18)
C10—C9—H9B	111.4 (18)	C3—C17—C16	109.37 (17)
C8—C9—H9B	110.3 (18)	C18—C17—C16	126.82 (18)
H9A—C9—H9B	106 (2)	C17—C18—C20	115.76 (18)
C9—C10—C16	112.16 (19)	C17—C18—C19	118.01 (18)
C9—C10—C11	114.6 (2)	C20—C18—C19	125.99 (18)
C16—C10—C11	106.56 (17)	C18—C19—C11	113.97 (18)
C9—C10—H10A	107.5 (14)	C18—C19—H19A	109.8 (18)
C16—C10—H10A	109.8 (14)	C11—C19—H19A	107.0 (18)
C11—C10—H10A	106.0 (14)	C18—C19—H19B	113.1 (19)
N12—C11—C10	107.31 (18)	C11—C19—H19B	107.2 (18)
N12—C11—C19	115.9 (2)	H19A—C19—H19B	105 (2)
C10—C11—C19	113.08 (19)	C21—C20—C18	120.57 (18)
N12—C11—H11A	104.9 (15)	C21—C20—H20A	118.2 (16)
C10—C11—H11A	109.1 (15)	C18—C20—H20A	121.2 (16)
C19—C11—H11A	106.1 (15)	C20—C21—C2	123.27 (19)
C13—N12—C14	108.0 (2)	C20—C21—H21A	118.1 (15)
C13—N12—C11	112.6 (2)	C2—C21—H21A	118.5 (15)
			
C10—C11—N12—C13	178.5 (2)		

### (I) (4R,4aR,7aR,12bS)-9-Hydroxy-3-methyl-1,2,4,4a,5,6,7a,13-octahydro-4,12-methanobenzofuro[3,2-e]isoquinolin-7-one] Hydrogen-bond geometry (Å, º)

**Table:**

*D*—H···*A*	*D*—H	H···*A*	*D*···*A*	*D*—H···*A*
O1—H1*A*···N12^i^	0.91 (4)	1.89 (4)	2.796 (3)	171 (3)

Symmetry code: (i) *x*+1, *y*, *z*.

### (II) (4R,4aR,7aR,12bS)-9-hydroxy-3-methyl-1,2,4,4a,5,6,7a,13-octahydro-4,12-methanobenzofuro[3,2-e]isoquinoline-7-one Crystal data

**Table:**

C_17_H_19_NO_3_	*D*_x_ = 1.388 Mg m^−^^3^
*M**_r_* = 285.33	Melting point < 550.2 K
Orthorhombic, *P*2_1_2_1_2_1_	Mo *K*α radiation, λ = 0.71073 Å
*a* = 8.8802 (6) Å	Cell parameters from 7368 reflections
*b* = 10.6208 (8) Å	θ = 0.4–32.6°
*c* = 14.4733 (9) Å	µ = 0.10 mm^−^^1^
*V* = 1365.05 (16) Å^3^	*T* = 296 K
*Z* = 4	Block, colorless
*F*(000) = 608	0.40 × 0.32 × 0.22 mm

### (II) (4R,4aR,7aR,12bS)-9-hydroxy-3-methyl-1,2,4,4a,5,6,7a,13-octahydro-4,12-methanobenzofuro[3,2-e]isoquinoline-7-one Data collection

**Table:**

Bruker KappaCCD diffractometer	4693 reflections with *I* > 2σ(*I*)
Radiation source: fine-focus sealed tube	*R*_int_ = 0.022
Horizonally mounted graphite crystal monochromator	θ_max_ = 32.6°, θ_min_ = 3.8°
CCD scans	*h* = −13→13
15227 measured reflections	*k* = −16→16
4920 independent reflections	*l* = −21→16

### (II) (4R,4aR,7aR,12bS)-9-hydroxy-3-methyl-1,2,4,4a,5,6,7a,13-octahydro-4,12-methanobenzofuro[3,2-e]isoquinoline-7-one Refinement

**Table:**

Refinement on *F*^2^	Primary atom site location: difference Fourier map
Least-squares matrix: full	Secondary atom site location: difference Fourier map
*R*[*F*^2^ > 2σ(*F*^2^)] = 0.033	Hydrogen site location: mixed
*wR*(*F*^2^) = 0.095	H atoms treated by a mixture of independent and constrained refinement
*S* = 1.07	*w* = 1/[σ^2^(*F*_o_^2^) + (0.0623*P*)^2^ + 0.0509*P*] where *P* = (*F*_o_^2^ + 2*F*_c_^2^)/3
4920 reflections	(Δ/σ)_max_ = 0.011
257 parameters	Δρ_max_ = 0.27 e Å^−^^3^
0 restraints	Δρ_min_ = −0.12 e Å^−^^3^

### (II) (4R,4aR,7aR,12bS)-9-hydroxy-3-methyl-1,2,4,4a,5,6,7a,13-octahydro-4,12-methanobenzofuro[3,2-e]isoquinoline-7-one Special details

**Table:**

Geometry. All esds (except the esd in the dihedral angle between two l.s. planes) are estimated using the full covariance matrix. The cell esds are taken into account individually in the estimation of esds in distances, angles and torsion angles; correlations between esds in cell parameters are only used when they are defined by crystal symmetry. An approximate (isotropic) treatment of cell esds is used for estimating esds involving l.s. planes.

### (II) (4R,4aR,7aR,12bS)-9-hydroxy-3-methyl-1,2,4,4a,5,6,7a,13-octahydro-4,12-methanobenzofuro[3,2-e]isoquinoline-7-one Fractional atomic coordinates and isotropic or equivalent isotropic displacement parameters (Å^2^)

**Table:**

	*x*	*y*	*z*	*U*_iso_*/*U*_eq_	
O1	0.93240 (13)	0.77706 (10)	0.49050 (9)	0.0470 (3)	
H1	1.015 (3)	0.792 (2)	0.517 (2)	0.069 (8)*	
C2	0.94522 (14)	0.66225 (11)	0.44868 (8)	0.0314 (2)	
C3	0.82077 (12)	0.60798 (11)	0.40680 (7)	0.02819 (19)	
O4	0.67658 (10)	0.65856 (9)	0.39772 (7)	0.03425 (18)	
C5	0.60923 (12)	0.58599 (11)	0.32226 (8)	0.0299 (2)	
H5	0.500 (2)	0.586 (2)	0.3283 (14)	0.039 (4)*	
C6	0.64963 (14)	0.64991 (13)	0.23020 (10)	0.0362 (2)	
O7	0.68419 (15)	0.76008 (11)	0.22794 (10)	0.0516 (3)	
C8	0.6488 (2)	0.56607 (16)	0.14696 (10)	0.0455 (3)	
H8A	0.549 (3)	0.528 (2)	0.1420 (15)	0.048 (5)*	
H8B	0.681 (3)	0.615 (2)	0.0937 (18)	0.058 (6)*	
C9	0.75507 (16)	0.45421 (14)	0.16230 (8)	0.0366 (3)	
H9A	0.856 (2)	0.486 (2)	0.1784 (15)	0.048 (5)*	
H9B	0.759 (3)	0.396 (3)	0.1098 (19)	0.065 (7)*	
C10	0.69690 (13)	0.37489 (11)	0.24255 (7)	0.0289 (2)	
H10	0.601 (2)	0.3431 (18)	0.2251 (13)	0.035 (4)*	
C11	0.79909 (14)	0.26306 (11)	0.26841 (8)	0.0322 (2)	
H11	0.810 (2)	0.2131 (17)	0.2157 (12)	0.033 (4)*	
N12	0.71416 (14)	0.18458 (10)	0.33458 (8)	0.0359 (2)	
C13	0.7868 (2)	0.06280 (15)	0.35203 (14)	0.0513 (4)	
H13A	0.8072	0.0217	0.2943	0.075 (8)*	
H13B	0.8796	0.0760	0.3847	0.090 (9)*	
H13C	0.7212	0.0110	0.3885	0.081 (8)*	
C14	0.68139 (17)	0.24994 (12)	0.42125 (9)	0.0367 (2)	
H14A	0.617 (3)	0.1946 (18)	0.4586 (14)	0.044 (5)*	
H14B	0.769 (3)	0.2710 (19)	0.4580 (15)	0.046 (5)*	
C15	0.59734 (14)	0.37270 (12)	0.40416 (8)	0.0323 (2)	
H15A	0.496 (2)	0.3593 (17)	0.3840 (13)	0.034 (4)*	
H15B	0.592 (2)	0.4201 (18)	0.4616 (15)	0.044 (5)*	
C16	0.67804 (11)	0.45335 (10)	0.33092 (7)	0.02580 (18)	
C17	0.83077 (12)	0.49110 (10)	0.36541 (7)	0.02626 (19)	
C18	0.96269 (12)	0.42399 (11)	0.35670 (8)	0.02859 (19)	
C19	0.95925 (15)	0.30434 (12)	0.30011 (10)	0.0356 (2)	
H19A	1.020 (3)	0.318 (2)	0.2452 (17)	0.055 (6)*	
H19B	1.006 (3)	0.233 (3)	0.3363 (19)	0.071 (7)*	
C20	1.09004 (13)	0.47869 (12)	0.39737 (8)	0.0325 (2)	
H20	1.191 (2)	0.4392 (19)	0.3896 (13)	0.039 (4)*	
C21	1.07953 (14)	0.59403 (12)	0.44358 (8)	0.0334 (2)	
H21	1.170 (2)	0.6333 (17)	0.4688 (15)	0.043 (5)*	

### (II) (4R,4aR,7aR,12bS)-9-hydroxy-3-methyl-1,2,4,4a,5,6,7a,13-octahydro-4,12-methanobenzofuro[3,2-e]isoquinoline-7-one Atomic displacement parameters (Å^2^)

**Table:**

	*U*^11^	*U*^22^	*U*^33^	*U*^12^	*U*^13^	*U*^23^
O1	0.0399 (5)	0.0442 (5)	0.0569 (6)	−0.0038 (4)	−0.0079 (5)	−0.0219 (5)
C2	0.0304 (5)	0.0351 (5)	0.0289 (5)	−0.0049 (4)	−0.0020 (4)	−0.0040 (4)
C3	0.0249 (4)	0.0316 (5)	0.0281 (4)	−0.0005 (4)	0.0002 (4)	−0.0050 (4)
O4	0.0277 (4)	0.0350 (4)	0.0400 (4)	0.0037 (3)	−0.0007 (3)	−0.0119 (3)
C5	0.0232 (4)	0.0327 (5)	0.0337 (5)	0.0021 (3)	−0.0005 (4)	−0.0050 (4)
C6	0.0263 (5)	0.0402 (6)	0.0421 (6)	0.0046 (4)	−0.0030 (4)	0.0064 (5)
O7	0.0452 (6)	0.0432 (6)	0.0663 (7)	−0.0039 (5)	−0.0079 (5)	0.0138 (5)
C8	0.0511 (8)	0.0538 (8)	0.0316 (5)	0.0087 (7)	−0.0034 (5)	0.0080 (5)
C9	0.0400 (6)	0.0445 (6)	0.0254 (4)	0.0030 (5)	0.0035 (4)	−0.0002 (4)
C10	0.0285 (5)	0.0334 (5)	0.0248 (4)	−0.0008 (4)	0.0007 (3)	−0.0048 (3)
C11	0.0352 (5)	0.0305 (5)	0.0310 (5)	0.0001 (4)	0.0044 (4)	−0.0064 (4)
N12	0.0415 (6)	0.0285 (4)	0.0378 (5)	−0.0021 (4)	0.0036 (4)	−0.0025 (4)
C13	0.0601 (10)	0.0338 (6)	0.0602 (9)	0.0061 (6)	0.0052 (7)	0.0034 (6)
C14	0.0438 (6)	0.0357 (6)	0.0306 (5)	−0.0041 (5)	0.0038 (5)	0.0024 (4)
C15	0.0314 (5)	0.0369 (5)	0.0285 (4)	−0.0044 (4)	0.0065 (4)	−0.0038 (4)
C16	0.0228 (4)	0.0295 (4)	0.0251 (4)	−0.0013 (3)	0.0012 (3)	−0.0042 (3)
C17	0.0237 (4)	0.0291 (4)	0.0260 (4)	−0.0012 (3)	−0.0001 (3)	−0.0029 (3)
C18	0.0248 (4)	0.0308 (5)	0.0301 (4)	0.0018 (4)	0.0008 (3)	0.0003 (4)
C19	0.0296 (5)	0.0331 (5)	0.0441 (6)	0.0039 (4)	0.0046 (5)	−0.0060 (4)
C20	0.0240 (4)	0.0387 (5)	0.0350 (5)	0.0014 (4)	−0.0020 (4)	0.0045 (4)
C21	0.0272 (5)	0.0412 (6)	0.0320 (5)	−0.0054 (4)	−0.0056 (4)	0.0014 (4)

### (II) (4R,4aR,7aR,12bS)-9-hydroxy-3-methyl-1,2,4,4a,5,6,7a,13-octahydro-4,12-methanobenzofuro[3,2-e]isoquinoline-7-one Geometric parameters (Å, º)

**Table:**

O1—C2	1.3660 (15)	C11—C19	1.5574 (18)
O1—H1	0.84 (3)	C11—H11	0.934 (17)
C2—C3	1.3860 (15)	N12—C14	1.4629 (17)
C2—C21	1.3975 (18)	N12—C13	1.467 (2)
C3—C17	1.3813 (14)	C13—H13A	0.9600
C3—O4	1.3948 (14)	C13—H13B	0.9600
O4—C5	1.4643 (14)	C13—H13C	0.9600
C5—C6	1.5379 (18)	C14—C15	1.5225 (19)
C5—C16	1.5407 (16)	C14—H14A	0.98 (2)
C5—H5	0.98 (2)	C14—H14B	0.97 (2)
C6—O7	1.2101 (18)	C15—C16	1.5398 (15)
C6—C8	1.498 (2)	C15—H15A	0.958 (19)
C8—C9	1.533 (2)	C15—H15B	0.97 (2)
C8—H8A	0.97 (2)	C16—C17	1.4998 (14)
C8—H8B	0.97 (2)	C17—C18	1.3770 (15)
C9—C10	1.5249 (17)	C18—C20	1.4010 (16)
C9—H9A	0.98 (2)	C18—C19	1.5122 (16)
C9—H9B	0.98 (3)	C19—H19A	0.97 (2)
C10—C16	1.5356 (14)	C19—H19B	1.01 (3)
C10—C11	1.5409 (17)	C20—C21	1.3988 (18)
C10—H10	0.954 (19)	C20—H20	1.00 (2)
C11—N12	1.4767 (16)	C21—H21	0.98 (2)
			
C2—O1—H1	107.5 (18)	C13—N12—C11	112.62 (12)
O1—C2—C3	119.90 (11)	N12—C13—H13A	109.5
O1—C2—C21	123.87 (11)	N12—C13—H13B	109.5
C3—C2—C21	116.22 (10)	H13A—C13—H13B	109.5
C17—C3—C2	120.81 (11)	N12—C13—H13C	109.5
C17—C3—O4	111.37 (9)	H13A—C13—H13C	109.5
C2—C3—O4	127.81 (10)	H13B—C13—H13C	109.5
C3—O4—C5	104.03 (8)	N12—C14—C15	111.38 (10)
O4—C5—C6	108.58 (10)	N12—C14—H14A	107.6 (12)
O4—C5—C16	104.99 (9)	C15—C14—H14A	108.4 (12)
C6—C5—C16	112.43 (9)	N12—C14—H14B	114.9 (13)
O4—C5—H5	109.9 (12)	C15—C14—H14B	106.6 (12)
C6—C5—H5	108.1 (12)	H14A—C14—H14B	107.7 (17)
C16—C5—H5	112.8 (13)	C14—C15—C16	111.10 (10)
O7—C6—C8	123.66 (14)	C14—C15—H15A	112.6 (11)
O7—C6—C5	120.62 (14)	C16—C15—H15A	108.1 (11)
C8—C6—C5	115.67 (11)	C14—C15—H15B	109.2 (12)
C6—C8—C9	109.94 (11)	C16—C15—H15B	108.9 (12)
C6—C8—H8A	107.9 (13)	H15A—C15—H15B	106.8 (17)
C9—C8—H8A	104.6 (13)	C17—C16—C10	108.88 (9)
C6—C8—H8B	108.7 (15)	C17—C16—C15	109.91 (9)
C9—C8—H8B	110.4 (15)	C10—C16—C15	108.80 (9)
H8A—C8—H8B	115 (2)	C17—C16—C5	98.12 (8)
C10—C9—C8	109.26 (11)	C10—C16—C5	118.14 (9)
C10—C9—H9A	108.6 (13)	C15—C16—C5	112.34 (9)
C8—C9—H9A	109.0 (13)	C18—C17—C3	124.03 (10)
C10—C9—H9B	104.8 (15)	C18—C17—C16	126.91 (10)
C8—C9—H9B	113.6 (16)	C3—C17—C16	109.05 (9)
H9A—C9—H9B	111.5 (19)	C17—C18—C20	115.70 (10)
C9—C10—C16	111.81 (10)	C17—C18—C19	117.86 (10)
C9—C10—C11	114.29 (10)	C20—C18—C19	126.32 (10)
C16—C10—C11	106.27 (9)	C18—C19—C11	114.49 (9)
C9—C10—H10	107.3 (11)	C18—C19—H19A	107.6 (14)
C16—C10—H10	108.4 (11)	C11—C19—H19A	108.1 (15)
C11—C10—H10	108.6 (12)	C18—C19—H19B	110.0 (16)
N12—C11—C10	106.97 (10)	C11—C19—H19B	108.4 (16)
N12—C11—C19	115.74 (11)	H19A—C19—H19B	108 (2)
C10—C11—C19	113.10 (9)	C21—C20—C18	120.67 (11)
N12—C11—H11	105.2 (11)	C21—C20—H20	118.9 (12)
C10—C11—H11	107.5 (11)	C18—C20—H20	120.3 (12)
C19—C11—H11	107.8 (12)	C2—C21—C20	122.43 (11)
C14—N12—C13	110.99 (12)	C2—C21—H21	117.6 (11)
C14—N12—C11	112.94 (9)	C20—C21—H21	119.8 (12)
			
C10—C11—N12—C13	169.5 (2)		

### (II) (4R,4aR,7aR,12bS)-9-hydroxy-3-methyl-1,2,4,4a,5,6,7a,13-octahydro-4,12-methanobenzofuro[3,2-e]isoquinoline-7-one Hydrogen-bond geometry (Å, º)

**Table:**

*D*—H···*A*	*D*—H	H···*A*	*D*···*A*	*D*—H···*A*
O1—H1···O4^i^	0.84 (3)	1.96 (3)	2.791 (2)	167 (3)

Symmetry code: (i) *x*+1/2, −*y*+3/2, −*z*+1.
